# Toner Lectures

**Published:** 1873-06

**Authors:** 


### Toner Lectures.

A series of lectures has been instituted at Washington under the "Toner Fund to encourage the discovery of new truths for the advancement of Medicine." The trustees of the fund are the following distinguished gentlemen.

Joseph Henry, L. L. D., Secretary Smithsonian Institution.

Joseph K. Barnes, M. D., Surgeon General, U. S. A.

J. C. Palmer, M. D., Surgeon General, U. S. N.

James E. Morgan, M. D., President Medical Society, D. C.

J. M. Toner, M. D., President American Medical Association.

The first lecture of this course was delivered at Washington, by Dr. J. J. Woodward, U. S. A., March 28th, upon the subject of "The Structure of Cancerous Tumors, and the manner in which adjacent parts are invaded." The lecture was illustrated by some seventy photographic screen illustrations and was successful. The second of the course was given by Dr. C. E. Brown-Sequard, his subject being "Nervous Force; the extent, variety, and power of its manifestations." The *Philadelphia Reporter* gives the following abstract of his lecture:

He said that in fact all disturbances of the human system were attributable to nervous influences. Even itch and other diseases of the skin could be traced to this same course, and the medical profession would never be able to treat diseases with anything like intelligence until this was fully understood. Epilepsy is always produced by the irritation of some peculiar nerve, and to be cured that nerve must be found and the cure reached through that. Physicians made many mistakes in attributing diseases to causes that had nothing to do with the matter. For instance, bed-sores, which were considered by medical men to be caused by pressure and the irritation produced by the excrement, were solely due to nervine influences. This he had proved by experiments on animals, and again many diseases of the limbs and joints which commonly were attributed to some local cause, were to be traced to the base of the brain. This he had proved beyond the peradventure of a doubt by experimenting on animals. He had known the knee joint to swell to three times its size in a single night from a disease of the spinal cord. The same was true in many of the lung diseases. Amputation of the limbs will produce an atrophy of medulla oblongata, by cutting off so much of the nervine nutrition. The nervous irritation would affect different people in different ways; for instance, one thousand people might come out of a hot crowded theatre into a cold storm, nine hundred of them escape injury, but of the one hundred injured, no two would be affected alike, but all would be affected through the same channel, the nerves of the skin.

